# Identifying where hospital and community trusts are managing general practices in England: a service mapping study

**DOI:** 10.3399/BJGPO.2023.0173

**Published:** 2024-09-04

**Authors:** Charlotte Davies, Catherine L Saunders, Fifi Olumogba, Manbinder Sidhu, Jon Sussex

**Affiliations:** 1 RAND Europe Community Interest Company, Cambridge, UK; 2 Department of Public Health and Primary Care, University of Cambridge, Cambridge, UK; 3 Health Services Management Centre, University of Birmingham, Birmingham, UK

**Keywords:** Delivery of healthcare, integrated care, primary healthcare, general practitioners

## Abstract

**Background:**

Organisations providing secondary care in the NHS in England have historically not also provided primary health care, but this is changing. Data on where this kind of ‘vertical integration’ is happening are lacking, making it difficult to evaluate its impact.

**Aim:**

To compile a comprehensive list of instances of secondary care trusts running general practices in England, to enable evaluation of the impact of such arrangements.

**Design & setting:**

This service mapping study comprises review, collation, synthesis, and analysis of published information describing secondary and primary care provision in the NHS in England in March 2021.

**Method:**

Desk-based collection, including hand-searching, of secondary care organisations’ statutory annual reports. Triangulation via comparison with national data on general practices, the general practice workforce and practice contracts.

**Results:**

It was possible to construct a database of all instances of trusts running general practices in England as of 31 March 2021. We have identified 26 trusts running a total of 85 general practices, operating across a total of 116 practice sites. These practices have on average fewer patients and fewer GP full-time equivalents than other general practices, and before becoming vertically integrated were performing less well in the Quality and Outcomes Framework.

**Conclusion:**

We recommend that national statistics recording the details of general practices contracting with the NHS should include whether each practice is owned by another organisation and whether that is an NHS trust, another public body, or a private organisation. Such data are required to enable evaluation of the impacts of this kind of vertical integration.

## How this fits in

Examples of hospitals running general medical (primary care) practices in the NHS in England are increasing. Where this type of vertical integration between primary and secondary care occurs, it is the result of local initiative, not mandated national policy. Until now, the full extent of such vertical integration was unknown. The present study rectifies that and describes the characteristics of vertically integrated practices.

## Introduction

A desire for better integration between primary and secondary healthcare has long existed in England, both to achieve better care for patients and greater efficiency in provision of that care.^1^ Numerous policy initiatives have aimed to achieve such integration.^2–6^ The sustainability of primary care is a subject of increasing concern and debate for policy makers in England.^7^ More integrated and coordinated services between primary and secondary care have been suggested to try to tackle workforce issues and help to meet growing patient demand on both primary and secondary healthcare services.^3^ In the NHS in England, the focus of policy has been on closer, more coordinated working between geographically proximate GP practices by the creation of primary care networks, a form of horizontal integration.^5,6^ A different approach has emerged from local initiatives, rather than national policy, in several places in England; namely, a form of vertical integration where a secondary care organisation, known in the NHS as a ‘trust’, manages one or more general practices, which deliver primary medical care to local populations and act as gatekeepers to secondary care.^8,9^ Similar types of vertically integrated arrangements have been tried in Spain and the USA.^10,11^ Other forms of vertical integration of patient care are possible, for example through shared access to medical records, but our focus is on instances where a secondary care trust is running primary care practices.

Vertical integration of this kind is a relatively new phenomenon in the NHS, but instances of it have been increasing since 2015. A qualitative evaluation undertaken in 2019/2020 highlighted that sustaining primary care in locations where practices were likely to close and better integration of care were the two main drivers of vertical integration between acute hospitals and GP practices in England and Wales.^8,9^ Prior to that study, there had been little systematic information on the rationale for, or desired impact of, vertical integration in an NHS setting, or on why it is developing in some locations despite not being required by NHS policy. A statistical study published since then found that vertical integration between an acute hospital and 10 general practices at one location in the West Midlands of England was associated with a reduction in the rate of unplanned hospital admissions and readmissions.^12^

There has until now been no comprehensive national review in England (or elsewhere, to our knowledge) of where and when the management of primary care providers by secondary care provider organisations has been implemented. Such data are required to enable evaluation of the impact of this kind of vertical integration, which in turn is an important question for policy on the organisation of health care provision. We have therefore undertaken such a review as part of a larger mixed-methods project to evaluate the impact of vertical integration on efficiency outcomes and patient experience. The current article presents our method and findings of where vertical integration of this kind had happened as of 31 March 2021, and describes some of the characteristics of the general practices involved. A full, detailed report of the wider research project of which this is part is published elsewhere.^13^

## Method

**Figure 1.**
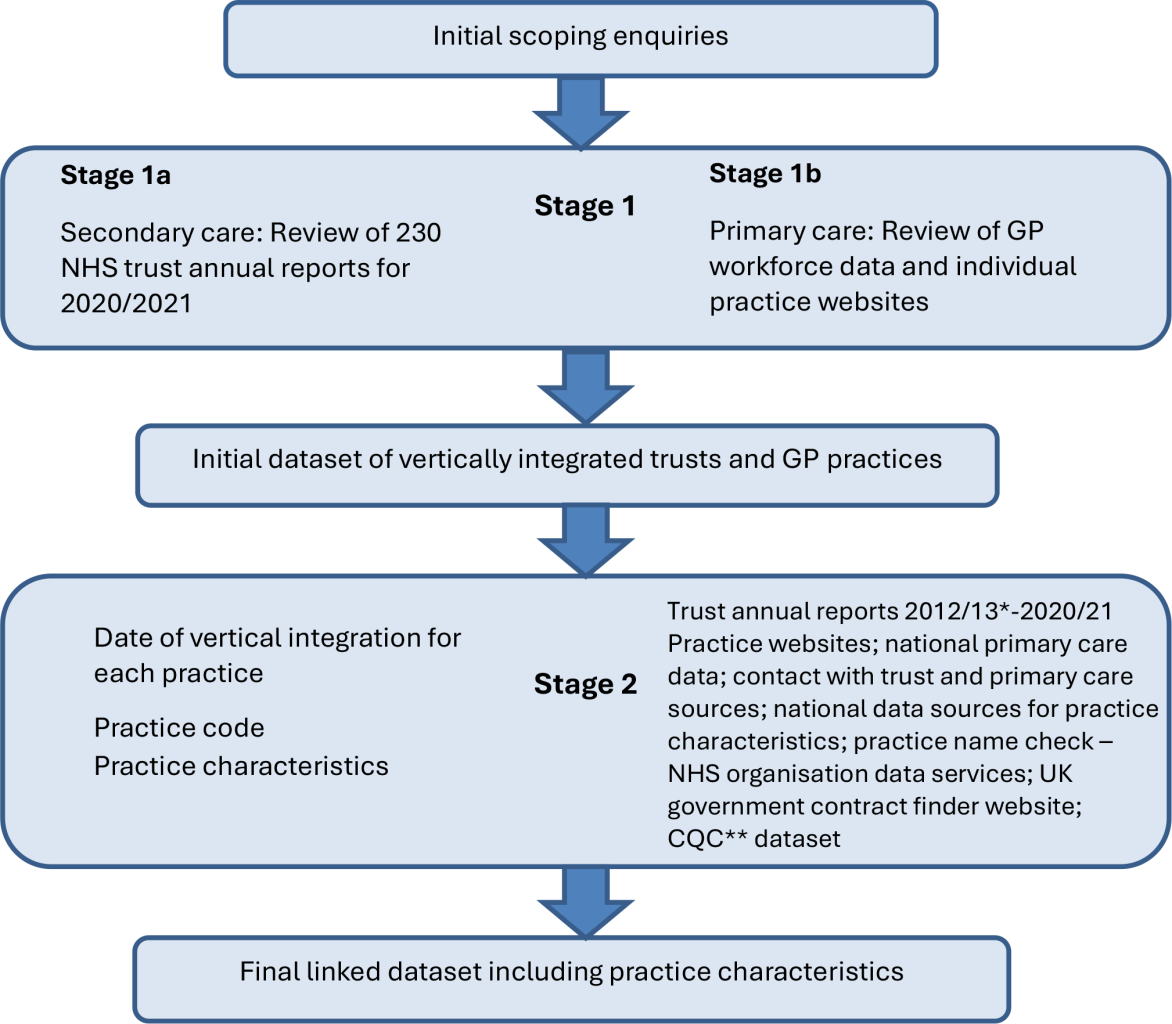
Flow chart of the search process. * Although vertical integration generally took place from 2015/16 onwards, we checked from 2012/13 for thoroughness. ** CQC = Care Quality Commission.

We undertook initial scoping work, including: an online search for data on where trusts managing general practices (which for the rest of this article we refer to as 'vertical integration' for brevity) was occurring in the NHS, and directly contacting national NHS and professional bodies that might hold those data (NHS England, Royal College of General Practitioners, and British Medical Association). This revealed that there was no organisation that held information systematically or completely on trusts and general practices in vertically integrated arrangements. We therefore carried out an in-depth search of multiple sources to identify where and when vertical integration has taken place in the NHS in England. Figure 1 summarises our scoping work.

We identified that the principal source of information on where (secondary care-providing) trusts run (primary care-providing) general practices, was the annual reports that trusts are required to publish. We hand searched the annual reports of all 230 NHS trusts listed by NHS Digital^14^ for the financial years 2019/20 and 2020/21. One member of the research team (FO) used the ‘find and retrieve’ search option for the terms “general practitioner”, “GP”, “general practice”, and “primary care” within each annual report and determined whether the trust had a financial or ownership relationship with a provider of GP services, and thus would be considered vertically integrated. For trusts with no vertically integrated practices, our expectation was that we would find less frequent use of the search terms in the report. All trust reports with identified vertically integrated GP practices and 20% of the remaining trust reports were then second reviewed by another member of the research team (CD) as a control. The original decisions were confirmed in all cases. We checked the website of each of the general practices identified as vertically integrated, to confirm their connection to the trust. We then (Stage 1b in Figure 1) inspected the General Practice Workforce data published by NHS Digital^15^ as at 31 March 2021, to identify practices where GPs with employment contracts categorised as ‘other’ were working and then check those practices’ websites. Such a categorisation is more common when a practice has novel ownership arrangements, for example when a trust is running a general practice and directly employs a GP.^9^

As a further robustness check we reviewed, on the UK government’s contract finder website,^16^ all new Alternative Provider Medical Services (APMS) contracts to identify whether a secondary care organisation held the contract. We also cross-checked our findings of vertically integrated practices with the Care Quality Commission register of general practices,^17^ filtered by the ‘Location primary inspection category’ and ‘Provider type/sector’. These checks identified no further instances of vertical integration.

We then returned to the annual reports of those trusts that had been found to be running GP practices to enable identification of practice-level details including the individual practice code. We sought to identify those general practices that were being run by a trust as at 31 March, so we excluded any general practices that might have been in such an arrangement previously but had exited it before the end of March 2021. This entailed starting with the trust’s annual report for 2020/21 and then working backwards, preceding year by preceding year, noting all mentions of GP practices or practice sites being vertically integrated (and whether they merged horizontally with other practices at any point; and whether they left the vertically integrated organisation at any date) until the date of the first vertical integration event (the first GP practice whose management was taken over by the trust). Where practice code, name, and date of integration were not all mentioned in the trust annual report, we visited individual practice websites, carried out online searching, and visited local and national media sources. We also used NHS Digital ‘GP and GP practice related data’ for GP practices and branch surgeries for 2 separate years (2022 and 2016) to identify practice codes.^18^ The final stage was to retrieve data on GP practice characteristics from NHS Digital data sources and link them to the identified vertically integrated practices.

## Results

During the search process, we identified five practices in England that were owned by trusts but which had been set up in that way specifically to deliver primary care to populations of people experiencing homelessness or to imprisoned persons, rather than to general populations of patients. These arrangements had been running for over 20 years. Our focus is on general practices that have changed to become vertically integrated with trusts, so we have excluded these five practices from the results presented and discussed below.

**Figure 2.**
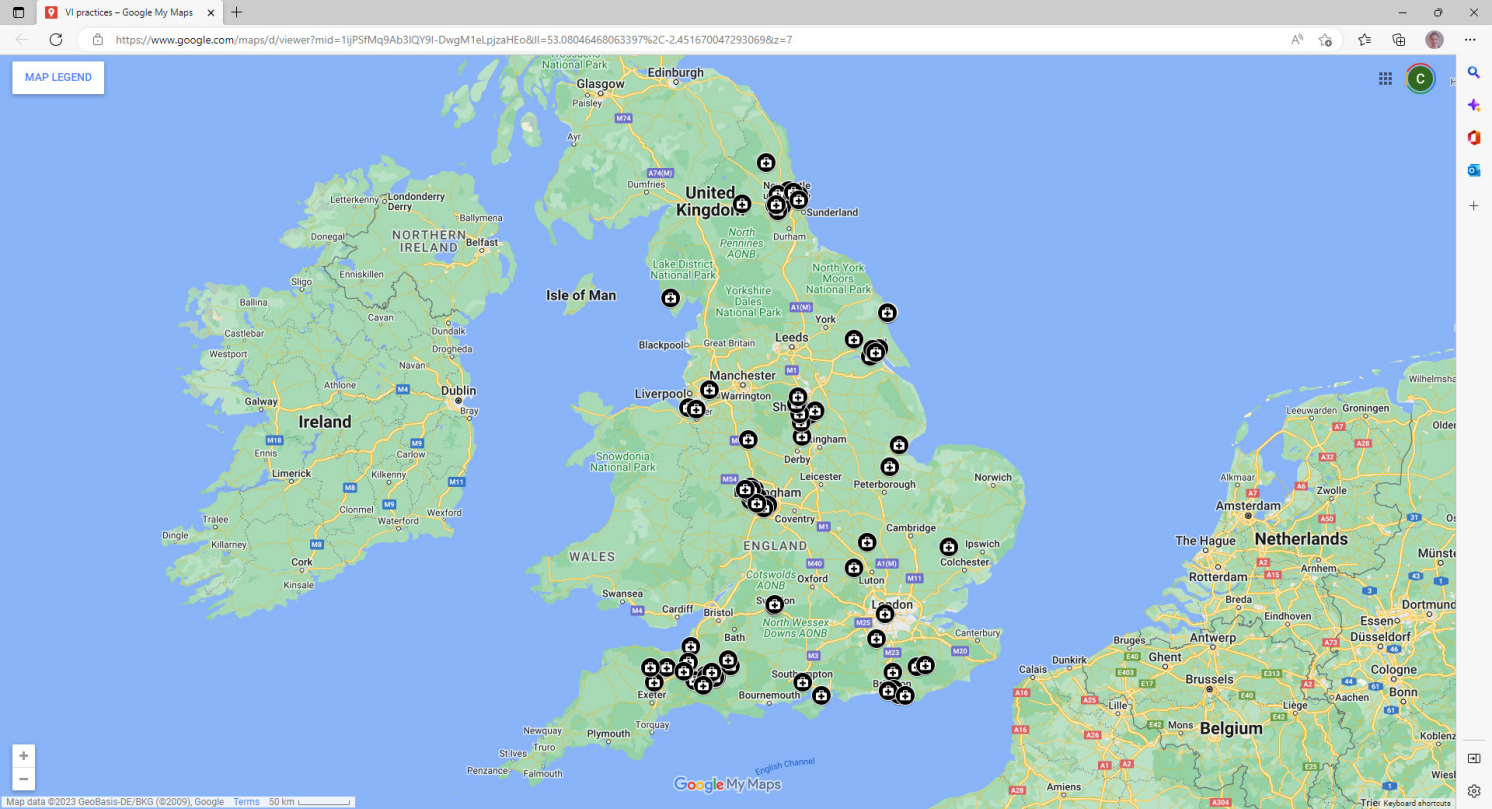
Locations of vertically integrated GP practices, England, March 2021.

As at the end of March 2021, 26 trusts were vertically integrated with 85 general practices, and those practices were operating from a total of 116 sites. Hence just 1.3% (*n* = 85/6576) of general practices in England were vertically integrated at that time, but 11.3% (*n* = 26/230) of trusts were involved. Fifteen of the trusts were running acute hospitals, and some of these were also providing secondary care community health services and/or mental health services. The remaining 11 trusts, which did not provide acute hospital services, we refer to hereafter as ‘community trusts’ for brevity, even though some of them also provide mental health services (and, indeed, one of them only provides mental health services). The 26 trusts are listed in Table 1, along with the number of general practices they each run, and when they each started running their first general practice. Figure 2 illustrates that the locations of the practices are spread widely across England.

**Table 1.** Vertically integrated trusts and numbers of GP practices, 31 March 2021

Trust name	Practice sites^a^	Unique practice codes^a^	Date first practice joined^b^ (Month-year)
**Acute hospital trusts:**			
Chesterfield Royal NHS Foundation Trust	7	3	May-15
Epsom and St Helier University Hospitals NHS Trust	1	1	Oct-19
Gateshead Health NHS Foundation Trust	4	4	Jan-21
Great Western Hospitals NHS Foundation Trust	2	2	Nov-19
Imperial College Health NHS Trust	1	1	Jul-16
Northumbria Healthcare NHS Foundation Trust	9	8	April-15
Royal Devon University Healthcare NHS Foundation Trust	1	1	Jan-18
Sandwell and West Birmingham Hospitals NHS Trust	7	3	May-19
Somerset Foundation NHS Trust	3	3	Sep-16
St Helens and Knowsley Teaching Hospitals NHS Trust	1	1	Apr-20
The Royal Wolverhampton NHS Trust	11	10	Jun-16
University Hospitals Birmingham NHS Foundation Trust	1	1	Jul-20
University Hospitals of Morecombe Bay NHS Foundation Trust	1	1	Oct-16
West Suffolk NHS Foundation Trust	1	1	Jun-20
Yeovil District Hospital NHS Foundation Trust	16	12	Apr-16
**Community trusts:**			
Cheshire and Wirral Partnership NHS Foundation Trust	4	3	Jul-15
Derbyshire Community Health Services NHS Foundation Trust	5	3	May-16
Dudley Integrated Health and Care NHS Trust	1	1	Apr-20
East London NHS Foundation Trust	5	2	May-13
Humber Teaching NHS Foundation Trust	9	8	Jun-15
Lincolnshire Community Health Services NHS Trust	3	2	Apr-19
North Staffordshire Combined Healthcare NHS Foundation Trust	2	1	Jun-18
Sheffield Health and Social Care NHS Foundation Trust	6	4	Jun-15
Solent NHS Trust	4	1	Oct-17
Southern Health NHS Foundation Trust	4	1	Apr-17
Sussex Community NHS Trust	7	7	Mar-19
**Totals (26 trusts**)	**116**	**85**	

^a^Excluding general practices specifically for patients experiencing homelessness or providing services for patients who are otherwise unable to access primary care. ^b^Dates identified from trust annual reports, practice websites, and local news media sources.

**Figure 3.**
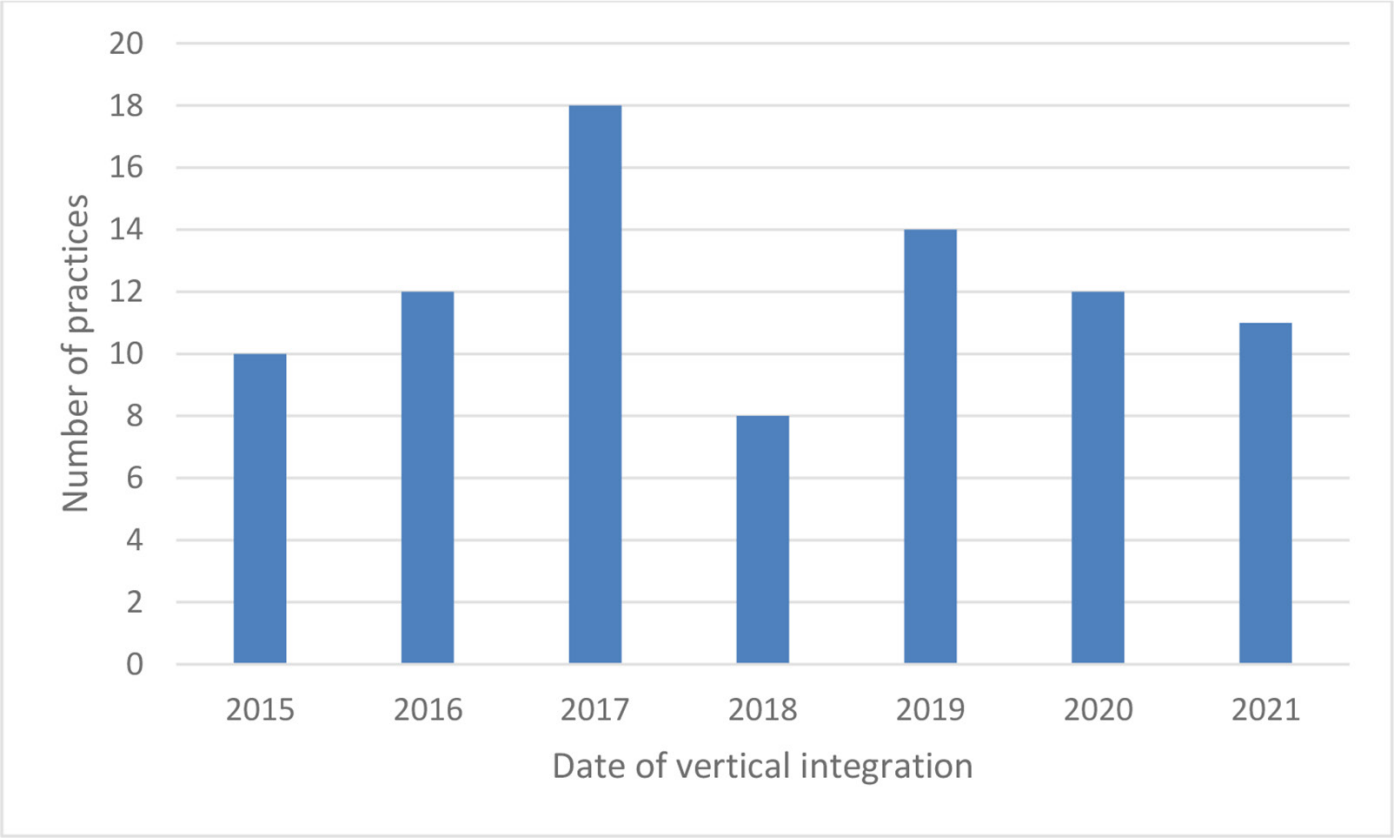
Date of vertical integration by number of practices.

The first case of vertical integration between a trust and a GP practice recorded in our dataset occurred in April 2015. Figure 3 shows the number of general practices becoming vertically integrated each year since 2015, with the annual total ranging between 8 and 18 (note that the 2021 figure only includes practices becoming vertically integrated before April that year). The mean number of practices run by each trust was 3.3 (median 2.5). The distribution is skewed with a handful of trusts each integrated with several general practices but many being integrated with only one or two practices (Table 1). The largest group of practices was run by an acute trust, which at 31 March 2021 was running 12 GP practices in South-West England.

Table 2 presents descriptive statistics (as at March 2021) comparing vertically integrated practices with other general practices, and disaggregating according to whether they are integrated with an acute trust or a community trust. A full list of the 85 general practices in England that were being managed by trusts as at March 2021 is provided in Table 3. On average, vertically integrated general practices have fewer patients than others, with a median list size of 6794 patients (mean 8902) compared to a median of 8029 patients (mean 9245) at other practices; and have fewer GPs, with a median 3.6 full-time equivalent (FTE) GPs compared to 4.3 FTE GPs at other practices. Practices vertically integrating with acute hospital trusts have slightly smaller list sizes on average than those combining with community trusts.

**Table 2.** Characteristics of practices, March 2021

Practice characteristic	Category	Vertically integrated			Not vertically integrated
		Total (*n* = 85)	Acute (*n* = 52)	Community (*n* = 33)	
Median patient list size (interquartile range)		6794 (5177–10 273)	6647 (5390–10 430)	6927 (4406–9507)	8028 (5195–11 734)
IMD, *n* (%) of practices in most deprived decile areas^a^		14 (16.5%)	6 (11.5%)	8 (24.2%)	986 (15.2%)
Contract type, *n* (%)^b^	APMS	12 (14.1%)	6 (11.5%)	6 (18.2%)	150 (2.3%)
	GMS	50 (58.8%)	33 (63.5%)	17 (51.5%)	4567 (70.4%)
	PMS	23 (27.1%)	13 (25.0%)	10 (30.3%)	1763 (27.2%)
Rural/urban, *n* (%)^c,d^	Rural	22 (27.5%)	18 (34.6%)	4 (14.3%)	1052 (16.6%)^e^
	Urban	58 (72.5%)	34 (65.4%)	24 (85.7%)	5302 (83.4%)^e^
QOF, *n* (%) achieving top 25% score^f^		50 (58.8%)	33 (63.5%)	17 (51.5%)	4904 (75.8%)^g^
Median GP FTEs (interquartile range)^h^		3.6 (2–6)	3.4 (2–5.5)	3.8 (2–6.1)	4.3 (2.3–7.2)

^a^IMD: The Index of Multiple Deprivation is the official measure of relative deprivation for small areas in England. It provides a rank from 1, which is the most deprived area, to 32,844, the least deprived area. We report the number of practices in the most deprived decile.

^b^Contract type: Alternative Provider Medical Services (APMS) contracts can be let to public and private sector; Personal Medical Services (PMS) contracts negotiated locally (with clinical commissioning groups in England); General Medical Services (GMS) contracts agreed nationally.

^c^Rural/urban: a classifier to define whether a practice is located in a rural or urban location (pre-specified geographically).

^d^Missing data *n* = 5, all from Vertically integrated Community practices.

^e^Missing data; denominator = 6355.

^f^QOF: Quality and Outcomes Framework indicator which is agreed as part of the NHS GP contract negotiations. We record the data for a practice achieving a QOF score within 25% of the maximum total quality points achievable.

^g^Missing data; denominator = 6470.

^h^GP FTE: Full-time equivalent GPs at the practice.

**Table 3.** Vertically integrated trusts and general practices, England, March 2021

NHS trust	Practice code	Trust type	Date of vertical integration
Cheshire and Wirral Partnership NHS Foundation Trust	N81117	Community	01/09/2019
Cheshire and Wirral Partnership NHS Foundation Trust	Y04664	Community	01/12/2017
Cheshire and Wirral Partnership NHS Foundation Trust	N81607	Community	01/07/2015
Chesterfield Royal NHS Foundation Trust	C81045	Acute	01/06/2019
Chesterfield Royal NHS Foundation Trust	C81008	Acute	01/07/2016
Chesterfield Royal NHS Foundation Trust	Y04995	Acute	01/05/2015
Derbyshire Community Health Services NHS Foundation Trust	C81638	Community	01/10/2016
Derbyshire Community Health Services NHS Foundation Trust	Y04977	Community	01/05/2016
Derbyshire Community Health Services NHS Foundation Trust	C81059	Community	01/05/2016
Dudley Integrated Health and Care NHS Trust	Y02653	Community	01/04/2020
East London NHS Foundation Trust	E81030	Community	01/04/2020
East London NHS Foundation Trust	E81044	Community	01/02/2020
Epsom and St Helier University Hospitals NHS Trust	H81618	Acute	01/10/2019
Gateshead Health NHS Foundation Trust	A85014	Acute	01/01/2021
Gateshead Health NHS Foundation Trust	A85620	Acute	01/01/2021
Gateshead Health NHS Foundation Trust	Y02658	Acute	01/01/2021
Gateshead Health NHS Foundation Trust	A85003	Acute	01/01/2021
Great Western Hospitals NHS Foundation Trust	J83035	Acute	01/11/2019
Great Western Hospitals NHS Foundation Trust	J83031	Acute	01/11/2019
Humber Teaching NHS Foundation Trust	B81014	Community	01/03/2021
Humber Teaching NHS Foundation Trust	B81653	Community	01/07/2020
Humber Teaching NHS Foundation Trust	B81060	Community	01/06/2020
Humber Teaching NHS Foundation Trust	B81052	Community	01/06/2018
Humber Teaching NHS Foundation Trust	B81006	Community	01/11/2017
Humber Teaching NHS Foundation Trust	Y02344	Community	01/06/2017
Humber Teaching NHS Foundation Trust	B81658	Community	01/06/2017
Humber Teaching NHS Foundation Trust	B81009	Community	01/06/2015
Imperial College Health NHS Trust	Y02589	Acute	01/07/2016
Lincolnshire Community Health Services NHS Trust	C83631	Community	01/04/2020
Lincolnshire Community Health Services NHS Trust	C83060	Community	01/04/2019
North Staffordshire Combined Healthcare NHS Foundation Trust	M83146	Community	01/06/2018
Northumbria Healthcare NHS Foundation Trust	A84619	Acute	01/06/2019
Northumbria Healthcare NHS Foundation Trust	A84045	Acute	01/04/2017
Northumbria Healthcare NHS Foundation Trust	A84002	Acute	01/02/2017
Northumbria Healthcare NHS Foundation Trust	A87002	Acute	01/04/2016
Northumbria Healthcare NHS Foundation Trust	A87006	Acute	01/04/2016
Northumbria Healthcare NHS Foundation Trust	A84025	Acute	01/04/2015
Northumbria Healthcare NHS Foundation Trust	A84007	Acute	01/04/2015
Northumbria Healthcare NHS Foundation Trust	A87004	Acute	01/04/2015
Royal Devon University Healthcare NHS Foundation Trust	L83052	Acute	01/01/2018
Sandwell and West Birmingham Hospitals NHS Trust	M88004	Acute	01/07/2019
Sandwell and West Birmingham Hospitals NHS Trust	Y02701	Acute	01/06/2019
Sandwell and West Birmingham Hospitals NHS Trust	Y06378	Acute	01/05/2019
Sheffield Health and Social Care NHS Foundation Trust	C88015	Community	01/06/2015
Sheffield Health and Social Care NHS Foundation Trust	Y05349	Community	01/06/2015
Sheffield Health and Social Care NHS Foundation Trust	M85713	Community	01/06/2015
Sheffield Health and Social Care NHS Foundation Trust	C88069	Community	01/06/2015
Solent NHS Trust	J82024	Community	01/04/2017
Somerset Foundation NHS Trust	L85609	Acute	01/04/2019
Somerset Foundation NHS Trust	L85052	Acute	01/04/2017
Somerset Foundation NHS Trust	L85038	Acute	01/09/2016
Southern Health NHS Foundation Trust	J82083	Community	01/04/2017
St Helens and Knowsley Teaching Hospitals NHS Trust	Y02510	Acute	01/04/2020
Sussex Community NHS Foundation Trust	G81043	Community	01/04/2021
Sussex Community NHS Foundation Trust	G81030	Community	01/04/2021
Sussex Community NHS Foundation Trust	H82044	Community	01/04/2021
Sussex Community NHS Foundation Trust	G81075	Community	01/04/2021
Sussex Community NHS Foundation Trust	G81061	Community	01/08/2020
Sussex Community NHS Foundation Trust	G81667	Community	01/07/2020
Sussex Community NHS Foundation Trust	G81083	Community	01/03/2019
The Royal Wolverhampton NHS Trust	M92014	Acute	01/11/2019
The Royal Wolverhampton NHS Trust	M92006	Acute	01/07/2018
The Royal Wolverhampton NHS Trust	M83132	Acute	01/02/2018
The Royal Wolverhampton NHS Trust	M92011	Acute	01/11/2017
The Royal Wolverhampton NHS Trust	M92028	Acute	01/09/2017
The Royal Wolverhampton NHS Trust	Y02757	Acute	01/07/2017
The Royal Wolverhampton NHS Trust	M92044	Acute	01/04/2017
The Royal Wolverhampton NHS Trust	M92042	Acute	01/01/2017
The Royal Wolverhampton NHS Trust	M92007	Acute	01/06/2016
The Royal Wolverhampton NHS Trust	M92002	Acute	01/06/2016
University Hospitals Birmingham	M85055	Acute	07/07/2020
University Hospitals of Morecombe Bay NHS Foundation Trust	A82033	Acute	01/10/2016
West Suffolk NHS Foundation Trust	D83064	Acute	01/06/2020
Yeovil District Hospital NHS Foundation Trust	L85048	Acute	01/04/2021
Yeovil District Hospital NHS Foundation Trust	L85056	Acute	01/03/2021
Yeovil District Hospital NHS Foundation Trust	L85003	Acute	01/04/2020
Yeovil District Hospital NHS Foundation Trust	L85032	Acute	01/02/2019
Yeovil District Hospital NHS Foundation Trust	L85007	Acute	01/12/2018
Yeovil District Hospital NHS Foundation Trust	L85061	Acute	01/11/2018
Yeovil District Hospital NHS Foundation Trust	L85026	Acute	01/05/2018
Yeovil District Hospital NHS Foundation Trust	L85027	Acute	01/12/2017
Yeovil District Hospital NHS Foundation Trust	L85064	Acute	01/08/2017
Yeovil District Hospital NHS Foundation Trust	L85004	Acute	01/07/2017
Yeovil District Hospital NHS Foundation Trust	L85010	Acute	01/04/2017
Yeovil District Hospital NHS Foundation Trust	L85066	Acute	01/04/2016

Vertically integrated practices are located in areas with similar levels of deprivation compared with other practices , with 16% (*n* = 14) of vertically integrated practices being in the most deprived decile of areas compared to 15% (*n* = 986) of other practices. It is noticeable that practices vertically integrated with community trusts are much more likely than those integrated with acute hospital trusts to be in the most deprived decile of areas.

Vertically integrated practices are considerably more likely than other practices to be on APMS contracts — only 2.3% (*n* = 150) of other practices have APMS contracts compared to 14.1% (*n* = 12) of vertically integrated practices — but GMS contracts are the most common contract type even among vertically integrated practices. Table 2 also shows a higher proportion of vertically integrated practices in rural areas (27.5%, *n* = 22) compared to other practices (16.6%, *n* = 1052). Vertically integrated practices are, on average, lower-achieving than others in terms of Quality and Outcomes Framework (QOF) scores, with only 58.8% (*n* = 50) of the vertically integrated practices achieving a score of at least 75% of the possible maximum, compared with 75.8% (*n* = 4904) of non-vertically integrated practices doing so. These findings are suggestive of practices with weaker performance being more likely to be vertically integrated. The direction of causation is not known, but an earlier study^8,9^ noted that an important rationale for practices to vertically integrate with a trust was because the practice was struggling to stay open due to financial and workforce-related challenges.

## Discussion

### Summary

Our work provides evidence of the extent of vertical integration in the NHS in England between trusts (providers of secondary care) and general practices (providers of primary care). At the end of March 2021, approximately one general practice in 80 was being run by a secondary care organisation, and one in nine such organisations were running one or more general practices. The number of general practices moving to a vertically integrated arrangement of this kind has been growing modestly but steadily since 2015. Hitherto, this has been the consequence of local initiative, not mandated by national policy.^8,9^ There was a flurry of interest by national policy makers and commentators in the England in early 2022, when the Secretary of State for Health at that time publicly expressed interest in trusts running general practices more widely.^19,20^ But, at the time of writing, vertical integration remains a matter to be determined locally. This is in line with the conclusions of the qualitative analysis by Sidhu and colleagues^8,9^ that vertical integration is an option worthy of consideration but it is not attractive to all practices or trusts. There are also various rationales for vertical integration, not least to support struggling practices as well as to enable better integration of patient care, and these are discussed elsewhere.^8,9^

We have used the term ‘vertical integration’ in this article as shorthand for the organisational arrangement where a secondary care trust is running one or more primary care practices. Other approaches to vertical integration are possible that do not entail direct management responsibility of this kind, though they may coincide with them, for example sharing of medical records. Those other approaches to vertical integration are not the subject of this article.

### Strengths and limitations

The strength of the results reported in this article is that they are the first, and so far only, comprehensive database of all vertically integrated general practices in the NHS in England. This provides a basis for quantitative and qualitative evaluation of vertical integration of trusts with general practices in England, such as the mixed-methods evaluation undertaken by a research team including the authors of this article and reported in detail elsewhere.^13^ That further evaluation analyses the impact of vertical integration on patients’ use of acute hospital services; how service delivery has changed or is expected to change; and the patient experience of vertical integration.

The process of identifying where vertical integration has taken place suffers from the limitation that it is labour-intensive to update, but it is nevertheless replicable. The approach we report is not without challenges. The annual reports published by every trust in the NHS in England provide the most comprehensive details of where vertical integration has taken place, but they do not always provide details of practice names, practice codes, and the date when the vertical integration started. Moreover, general practices may undergo several stages of reorganisation as part of the vertical integration process; the identification process is complicated where mergers between general practices (horizontal mergers) occur during the reorganisation that accompanies vertical integration.

### Comparison with existing literature

No other literature has attempted to identify all instances (in England) of secondary care providers running general practices.

### Implications for research and practice

We recommend that national statistics recording the details of general practices should in future include whether each practice is owned by another organisation and, if so, whether that organisation is an NHS trust, or another public body or private organisation. The result would be that, in future, there would be ready identification of all general practices’ ownership arrangements. Such data would support evaluation of the impacts of this kind of vertical integration.

## Data

The data are fully presented in the article tables. Source data, all from publicly available sources, are referenced.
